# Diagnostic Benefits and Surgical Implications of Methods for Tumor Localization in Sigmoid and Rectum Tumors

**DOI:** 10.3390/diagnostics14131363

**Published:** 2024-06-27

**Authors:** Mehmet Onur Gul, Mehmet Akcicek, Nidal Iflazoglu, Kadir Corbaci, Cuma Ali Emir, Mehmet Guzel, Cem Kaan Parsak

**Affiliations:** 1 Surgical Oncology Clinic, Malatya Training Research Hospital, 44000 Malatya, Turkey; drcumali@hotmail.com; 2 Faculty of Medicine, Department of Radiology, Malatya Turgut Özal University, 44000 Malatya, Turkey; mehmet.akcicek@ozal.edu.tr; 3 Surgical Oncology Clinic, Bursa City Hospital, 16110 Bursa, Turkey; nidal1933@yahoo.com; 4 General Surgery, Osmaneli Mustafa Selahattin Çetintaş State Hospital, 11500 Bilecik, Turkey; dr.kadircorbaci@gmail.com; 5 Gastroenterology Surgery, Malatya Training Research Hospital, 44000 Malatya, Turkey; mehmetguzel209@gmail.com; 6 Faculty of Medicine, Department of Surgical Oncology, Cukurova University, 01330 Adana, Turkey; cparsak@yahoo.com

**Keywords:** colon cancer, colonoscopy, computed tomography, rectum cancer, tumor localization

## Abstract

(1) Background: In our study, we aimed to determine the accuracy rates of imaging methods for sigmoid, rectosigmoid colon, and rectum cancer. (2) Methods: Patients with tumors located in the rectosigmoid colon, sigmoid colon, and rectum who were operated on were included. Upon admission, we examined the patients’ first diagnostic colonoscopies and their preoperative repeat control colonoscopies and computed tomography (CT) report. (3) Results: In this study, 23 patients (57.5%) were male. The overall accuracy rates were 80.0% (32/40) in colonoscopy, 65.0% (26/40) in preoperative CT, and 87.5% (35/40) in retro CT, and the differences among the examination methods were statistically significant (*p* = 0.049). The sensitivity levels decreased to 50.0% for colonoscopy and preoperative CT and 75.0% for retro CT in rectosigmoid colon tumors. In rectal tumors, the sensitivity levels were 75.0% in colonoscopy, 60.0% in preoperative CT, and 80.0% in retro CT. In two patients, the tumor location was given incorrectly, and postoperative pathological evaluations indicated T3N0 tumors; the initially planned treatment was thus changed to include radiotherapy in addition to chemotherapy in the postoperative period because the tumor was located in the middle rectum. (4) Conclusions: Accuracy in tumor localization in sigmoid, rectosigmoid, and rectum tumors still needs to be improved, which could be accomplished with prospective studies. CT evaluations for cancer localization in this patient group should be re-evaluated by a radiologist.

## 1. Introduction

Colorectal cancer is the fourth leading cause of cancer death and the third most common malignancy worldwide [1]. Based on colonoscopic examination and mostly contrast-enhanced tomographic evaluation, the tumor locations in colon tumor patients are determined, and surgical planning is performed. Today, colonoscopy is still considered the “gold standard” for the diagnosis of colorectal cancer (CRC) and the determination of the anatomical colon segment where the lesion is located [2,3]. However, especially in rectosigmoid colon tumors, sometimes this is insufficient to clearly determine the location of the tumor [2]. Some previous and current studies state that colonoscopic evaluation is the most effective method for performing tumor localization [4]. However, there are also studies that argue the opposite and emphasize that further evaluations are needed for more accurate colorectal tumor localization after colonoscopic examination [5]. The latter may need to be repeated for the operation, especially in the preoperative period. According to the literature, colonoscopic examination or imaging performed for colon tumors can sometimes be misleading and leave the surgeon and the patient unprepared for the disease. In their study, Borda et al. stated that colonoscopic tumor localization evaluation is better than tomography and that localization in the descending colon, cecum, and obstructive tumors is more difficult. In another study, the authors reported that both endoscopists and radiologists should not be overconfident in upper-rectal or low-sigmoidal cancer cases and that erroneous evaluations may occur [3]. In our study, the frequency of tumors with uncertain location, preoperative, and perioperative results was high in the rectum, sigmoid colon, and rectosigmoid colon. We designed this study to calculate the accuracy rates of imaging localization methods for adenocarcinomas in these regions.

## 2. Materials and Methods

**Figure 1:**
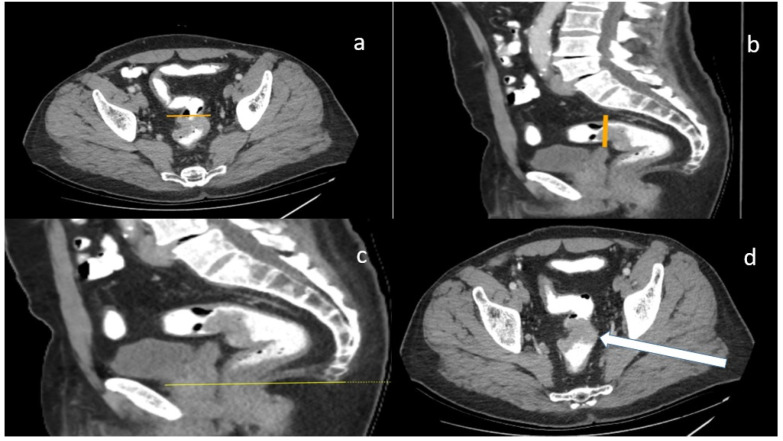
(**a**) Sigmoid take-off with axial plane (yellow line); (**b**) sigmoid take-off with sagittal plane (yellow line); (**c**) anorectal line with sagittal plane (yellow line); (**d**) tumor appearance with axial line (white arrow shows tumor).

Patients with tumors located in the rectosigmoid colon, sigmoid colon, and rectum who were operated on by Malatya Training and Research Hospital Surgical oncology doctors between January 2017 and January 2024 were included in our study. This study received the approval of the Ethics Committee of Turgut Özal University Malatya Training and Research Hospital (approval number E-30785963-020-220617). Only rectum and colon operations performed in our hospital by the authors’ team were included in the study. The rectosigmoid colon was considered the segment between the initial area of the sigmoid colon segment and the last Houston valve. The coiled segment of the sigmoid colon between 40 and 15 cm from the anus was considered the sigmoid colon. All tumors located in the segment from 4 cm from the entrance of the anus or 2 cm above the dentate line to the rectosigmoid area were considered rectum tumors (Figure 1) [6]. Anal canal tumors were excluded from the study.

Upon admission, we examined the patients’ first diagnostic flexible colonoscopies and preoperative control colonoscopies, if any, and calculated the number of patients with a significant change in the type of surgery or oncological treatment by subtracting the number of treatments planned after the initial presentation and that of the treatments required after surgery.

**Figure 2:**
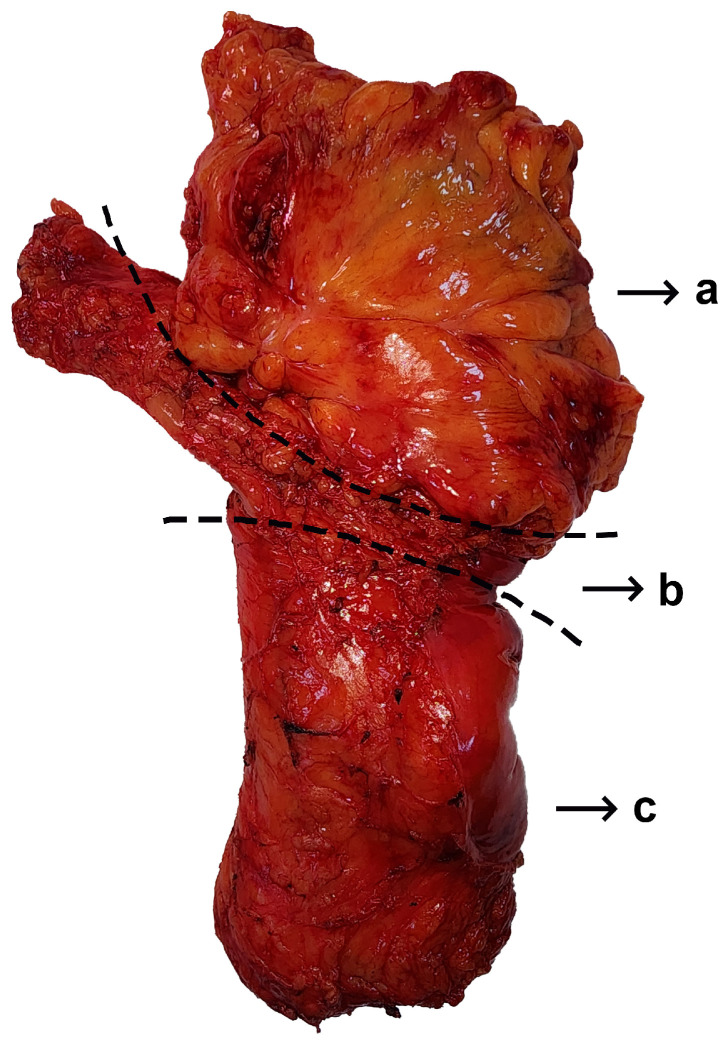
Middle-rectum tumor with total mesorectal excision view. a. Sigmoid colon marked area; b. Rectosigmoid area; c. Rectum area.

**Figure 3:**
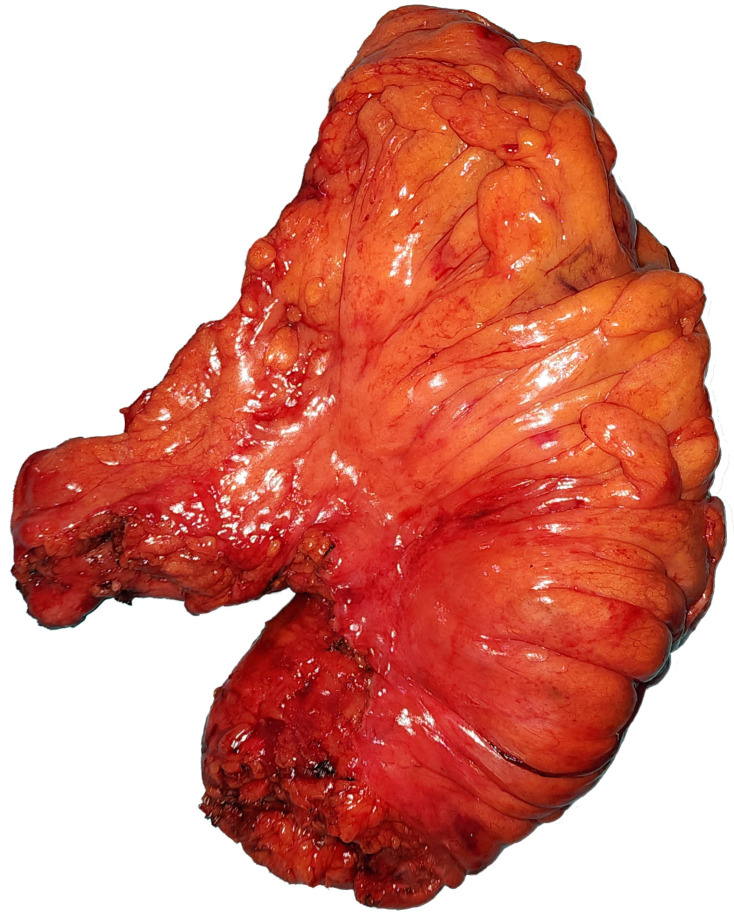
Sample image from a patient who underwent high–low anterior resection but for whom anterior resection was planned.

The histopathological evaluations of the patients were examined to ascertain the number of lymph nodes removed, the number of lymph node metastases, lymphovascular invasion, and perineural invasion status. Patients who did not have a colonoscopy at our hospital and were admitted to our clinic after having had a colonoscopy at an external center, who did not have a tomography at our hospital and underwent surgery after an external center’s tomography was evaluated, whose data were missing or uncertain in the information operating system, who underwent emergency surgery and only had a tomography scan, or who were operated on due to perforation without an imaging examination were excluded from the study. The rates were calculated without statistical evaluation, since control colonoscopies were performed primarily on patients whose tumor location was uncertain and whose number was insufficient. Since magnetic resonance imaging was not used in all patients, it was not included in the comparison. Anterior resection was performed on sigmoid colon tumors. In tumors in the part of the sigmoid colon close to the rectosigmoid region or in the rectosigmoid region, in addition to anterior resection, resection of the upper rectum (an area of approximately 5 cm) was performed to preserve the surgical margin. The operations of this patient group were coded as low anterior resections involving the upper rectum (Figure 2). In other rectum tumors, surgery involving total mesorectum was performed, whether or not neoadjuvant chemoradiotherapy was received by the patient (Figure 3). Loop ileostomy was performed in all patients who underwent total mesorectal excision, or coloanal or colorectal anastomosis.

### 2.1. CT Scanning Evaluation

All patients had computed tomography (CT) scans with contrast, and patients who were evaluated with CT without contrast or did not have a tomography were excluded from the study. The CT evaluations of the patients were performed at our hospital, or an external center due to service procurement; the CT scans taken for tumor patients were either evaluated through a discussion in the tumor council or discussed with radiologists experienced in abdominal tomography, and the patients were then taken into surgery. The preoperative contrast-enhanced tomography reports of the patients were re-examined by a single radiologist for the study, and tumor localization was defined in centimeters (cm) and location. This group was called retro CT evaluation. Axial CT scans were performed with a section thickness of 2.5 mm following the application of intravenous contrast material, using the 128 Slice GE Revolution EVO CT Scan multidetector device (General Healthcare, Chicago, IL, USA) after cleaning the patients’ bowels and making the necessary preparations. The axial images were processed on the workstation, and sagittal and coronal reformatted images were obtained.

The rectum, which has no haustral structures and is accompanied by the mesorectal layer, up to the rectosigmoid junction at the level of the 3rd sacral vertebra, from the anorectal angle, consists of the lower (up to 8 cm), middle (between 8–12 cm), and upper (12–15 cm) sections, all of which were examined. Sigmoid take-off was defined as the point at which the sigmoid moves horizontally away from the sacrum on the sagittal view MRI and as the point at which the sigmoid exits ventrally in the axial view. Axial, coronal, and sagittal CT images were used together to calculate the distance from the tumor to the anorectal junction. For segments that continued without angulation, the total distance was calculated by multiplying the section thickness, spacing, and number of sections, and for segments that were angulated and where a specific section was observed in a single section, this was performed by adding these distances together and measuring the entire length of the part included in the section.

### 2.2. Statistical Analysis

The data obtained in the study were analyzed by using the IBM SPSS Statistics for Windows version 29 program (Armonk, New york, NY, USA). Descriptive data were presented as numbers (n), rates (%), means, and standard deviation values. The tumor localization performed during surgery was considered the gold standard, and the sensitivity and specificity levels of colonoscopy, preoperative CT, and retro CT findings were calculated. Fisher–Freeman–Halton’s exact test was used to compare the localization accuracy rates of the examination methods according to the tumor regions. Cohen’s Kappa coefficient was used to evaluate compatibility between surgical locations and colonoscopy, preoperative CT, and re-read CT results. The chi-square test and Fisher’s exact test were used to examine the effects of individual and medical characteristics on the accuracy level of colonoscopy, preoperative CT, and retro CT findings. The statistical significance level was accepted when *p* < 0.05 in all analyses.

## 3. Results

In this study, 23 patients (57.5%) were male. Only one patient (2.5%) had a high-grade tubular adenoma, as per the preoperative pathology report, and the surgery was performed due to the presence of cancer. The remaining patients had adenocarcinoma (n = 39, 97.5%), and all specimen pathologies were reported as adenocarcinoma. The most frequently detected pathological stage after the operation was stage 2A (n = 18, 45.0%), followed by stage 1 (n = 11, 27.5%) and stage 3B (n = 6, 15.0%). A total of 48.7% (n = 19) of the tumors were well differentiated. Table 1 shows the demographic and clinicopathological characteristics of the patients. The mean largest tumor size was 45.2 mm (+SD 20.8). A total of 40% (n = 16) of the patients had lymphovascular invasion, 20% (n = 8) had perineural invasion, and 15% (n = 6) had tumor budding. A total of seven patients received neoadjuvant therapy (17.5%). Further, 42.5% (n = 17) of the surgeries were performed with open surgery and 57.5% (n = 23) with laparoscopic surgery. In 10% (n = 4) of cases, there were ileus findings, even though there was no complete obstruction before the operation, and the rate of patients who underwent ileostomy during the surgery was 52.5% (n = 21).

During surgery, 40% (n = 16) of colorectal tumors were detected in the sigmoid colon, 10% (n = 4) in the rectosigmoid region, and 50% (n = 20) in the rectum. The tumor localization results from both surgery and imaging evaluations are given in Table 2. As shown in Table 3, the sensitivity levels of the tests were highest in sigmoid colon tumors (93.8% for colonoscopy, 75.0% for preoperative CT, and 100.0% for retro CT). The sensitivity levels decreased to 50.0% for colonoscopy and preoperative CT and to 75.0% for retro CT in rectosigmoid colon tumors. In rectal tumors, the sensitivity levels were 75.0% for colonoscopy, 60.0% for preoperative CT, and 80.0% for retro CT. When comparing accuracy rates according to the tumor region, no statistically significant relationships were found for all three examination types (Table 4). With respect to surgery localization, the Kappa test showed agreement values of 0.675 for the colonoscopy findings, 0.791 for retro CT, and 0.491 for preoperative CT (all *p* < 0.001).

No statistically significant relationships were found in the analyses examining the effect of the individual and medical characteristics of the patients on the accuracy levels of tumor localization in colonoscopy, preoperative CT, and retro CT (Table 5). The overall accuracy rates were 80.0% (32/40) in colonoscopy, 65.0% (26/40) in preoperative CT, and 87.5% (35/40) in retro CT, and the differences among the examination methods were statistically significant (*p* = 0.049) (Table 6). In pairwise comparisons, the accuracy rate of retro CT was found to be higher than that of preoperative CT (*p* = 0.018). No significant difference was found in other pairwise comparisons. When the accuracy rates were evaluated according to tumor localization, there were no statistically significant differences among the examination methods. If we exclude tumors not seen/reported in the preoperative CT evaluation, the overall accuracy rate of preoperative CT increases to 81.3% (26/32), and the rates by location increase to 100% for the sigmoid colon, to 50% for the rectosigmoid colon, and to 75% for the rectum.

Patients with widespread metastases in the abdomen who were operated on for palliative treatment were excluded from the study; only one (2.5%) patient with a sigmoid colon tumor had two millimetric metastases in the pelvic region, and cytoreductive surgery and anterior resection were performed. In a patient with a sigmoid colon tumor (44-year-old male), due to a widespread family history of endometrium and colon cancer, total colectomy and ileorectal anastomosis were performed laparoscopically. Control endoscopy was performed before the operation in 13 (32.5%) patients; cancer in six of these patients was interpreted as rectum cancer; in three, as rectosigmoid cancer; and in four, as sigmoid colon cancer. All three rectosigmoid colon tumors that underwent control endoscopy were found to extend from the upper rectum to the middle rectum, and total mesorectal excision and loop ileostomy were performed in these patients. As an operational finding in these three patient groups, we noticed that the pelvic peritoneum adhered to the rectum serosa at the lower border of the middle rectum and that the area identified as reflection extended from a lower location.

Of the eight patients whose tumor localization evaluation results were different between colonoscopic or CT imaging and surgery, only five of them had the type of surgery changed in a way that changed the patient’s treatment. Although ileostomy was initially considered unlikely in these two patients, it was performed in all of them after the operation. In addition, in two of these patients, cancer was postoperatively pathologically evaluated as T3N0, and the patients received radiotherapy in addition to chemotherapy in the postoperative period because the tumors were located in the middle rectum (Table 7).

## 4. Discussion

Correct lesion localization before surgery is one of the most important aspects of optimal preoperative surgical planning. Incorrect localization causes on-table changes in surgical treatment, especially in minimally invasive surgery, or leads to the need to place a different type of robotic/laparoscopic port [7,8]. Although no situation that could have compromised the surgical procedure of the patients or posed a life-threatening risk occurred in our study, in a total of five patients, the type of surgery was changed, and a more advanced operation was performed.

Although colonoscopy is considered the gold standard for detecting colorectal lesions, studies on its accuracy in lesion localization are still insufficient [9]. In addition, studies have reported that it is not clear whether computed tomography scans, which assist in tumor staging, can be considered an aid in accurately localizing colon lesions [8,10]. Various publications have shown variability in the accuracy of colonoscopy, ranging from 79% to 88% [2,8,11,12]. In contrast, few studies have investigated the accuracy of CT in determining lesion location and reported that overall accuracy ranged from 42.3% to 90.5% [3,10,13]. Lee et al., in a retrospective analysis of 104 patients with cancer located throughout the colon, reported accuracy rates of 79.8% for colonoscopy and 50% for CT and noted that lesions were missed in 32.7% of cases [10]. However, we noticed that in the above study, no information was given about how the tomography results had been evaluated and whether re-evaluation had been performed. Similarly, Feuerlein et al. analyzed data from 46 patients and showed that colonoscopy and CT imaging had accuracy rates of 78.7% and 67.4%, respectively, in localizing colon lesions [14]. In contrast, Loffeld et al. reported that colonoscopy and CT scans showed higher accuracy rates in localizing sigmoidal and rectal lesions compared with the literature [3]. Nayor et al. stated that errors in endoscopic colon cancer localization are high and emphasized that they occur especially in right-colon tumors [15]. Another study reported overall accuracy rates of 87.5% for colonoscopy and 90.5% for imaging. In a prospective, multicenter analysis of 79 patients with colorectal cancer, Johnstone et al. showed that with colonoscopy, 81% of tumors were accurately detected. The same study showed that based on CT, the primary tumor could not be detected in 23.1% of the cases and that the accuracy rate in cases where cancer was detected was 88.3% [16]. In a study conducted by Souza et al., it was investigated whether the measurement of the sigmoid colon in sample analysis can confirm the preoperative starting point to define the rectum. The authors of the study stated that the distance between the rectosigmoid area and the peritoneal reflection is approximately 2 cm. Furthermore, the study highlighted that no direct evaluation in the literature can distinguish and define the rectum sigmoid colon based on MRI, histopathological evaluation, and operation findings [17]. In our study, there were patients with errors in tumor localization despite multiple colonoscopic examinations, and the fact that numerous colonoscopies and re-evaluations of CT did not provide a complete result made us think that there were other factors explaining this situation. Although we supposed that this was due to different pelvic types in the patients and peritoneal reflection varying from person to person, we could not evaluate this because we did not have data. By performing rigid rectosigmoidoscopy during colorectal surgery, Neto et al. determined that peritoneal reflection was in a lower location in overweight patients and women who had given birth more than twice [18]. Yigun et al. reported that in women, the distance from the distal border of the anal canal to the anterior pelvic reflection was longer than in men [19]. It was reported that especially women who have given birth have larger pelvises than men and that giving birth may be one of the factors affecting this distance.

Based on data from 729 patients, in Manigrasso et al.’s study, colonoscopy and CT scans were accurate in 74.6% and 70.1% of cases, respectively. However, when only lesions detected based on CT were considered, this imaging tool’s accuracy in localizing colon lesions increased to 77.2% [6]. Elnaggar et al. reported that colonoscopy and contrast-enhanced computed tomography had equal accuracy rates in tumor localization in right-colon, splenic flexure, and descending colon tumors. However, only the rates were evaluated, and no statistical data were shared. It was also emphasized in the same study that colonoscopy is superior in rectal cancer detection [20]. In our study, the overall accuracy rates were found to be 80.0% (32/40) in colonoscopy, 65.0% (26/40) in preoperative CT, 87.5% (35/40) in retro CT, and the differences among the examination methods were statistically significant (*p* = 0.049). In pairwise comparisons, the accuracy rate of retro CT was found to be higher than that of preoperative CT (*p* = 0.018).

The accurate localization of the tumor is critical for surgical planning, especially in laparoscopic surgery, where the surgeon cannot palpate the colon to detect the cancer mass. To avoid complications, many surgeons prefer to perform a repeat endoscopy when performed by the endoscopist who directed the initial colonoscopy. The CT staging of the abdomen/pelvis is a standard investigation in CRC patients; however, only a few studies have evaluated its utility in correcting localization errors and potentially eliminating the need for repeat endoscopy [3,10,21]. Despite years of research on the accuracy and utility of CT in correcting localization errors during the initial endoscopy, clear guidelines and definitions are limited [6,22]. The general application of the results of these studies is limited by small sample sizes and strict selection criteria, making it difficult to draw meaningful conclusions about the utility of this method. Not many studies have compared staging CT with re-evaluation and repeat endoscopy. Clinically, this is also important because repeat endoscopy has been shown to prevent the occurrence of localization errors, and many surgeons consider repeat endoscopy to be the standard of care for referred cases [21,23]. Therefore, surgeons may not consider repeating the colonoscopy if they see a CT mass compatible with the first colonoscopy result. Incorrect tumor localization may change the surgical procedure and the planned surgical strategy, including switching from laparoscopic surgery to open surgery, in 4–12% of cases [24,25]. Studies have reported that under certain circumstances, an error does not affect the surgical procedure (e.g., only right hemicolectomy rather than extended right hemicolectomy due to misdiagnosis between the cecum and ascending colon). However, various publications have shown that serious situations, such as the resection of a colon segment that does not contain a tumor, are due to previous incorrect cancer localization [10,26,27]. In their study on the causes of errors in the surgical localization of CRC in the proximal or distal segment, Borda et al. reported that the initial surgical plan had to be changed in only one case and that it was necessary to switch to open surgery and perform intraoperative colonoscopy in two cases [2]. This situation is not compatible with our study, and no problems developed in our study except for errors such as high–low anterior resection when anterior resection was planned or low anterior resection with complete mesorectal excision when high–low anterior resection was planned. There were no cases where the tumor was not removed in our study. 

Azin et al. confirmed that repeat endoscopy is superior to staging CT as a diagnostic tool to correct localization errors derived from the initial endoscopy [28], with overall sensitivity rates of 80.8% for repeat endoscopy and 42.3% for CT staging (*p* < 0.01). They reported that adding repeat endoscopy to staging CT following diagnostic endoscopy was associated with fewer location-based surgical changes in the surgical plan and complications. Although the number of repeated endoscopy operations in our study was limited, employing this method could not provide accurate tumor locations in three patients whose previous imaging was incompatible with surgery. Accuracy in preoperative tumor localization in colorectal cancer is essential for adequate resection and treatment planning. In their review, Acuna et al. found that the rate of localization errors was higher in traditional colonoscopy (15.4%) than in colonoscopic marking (9.5%), which was deemed to be safer and to cause fewer errors [7]. This discrepancy underscores the need for improved localization techniques, especially with the increasing adoption of laparoscopic resection for colorectal cancer. Therefore, colonoscopic tattooing has been recommended for routine use due to its lower error rate and safety profile. This significant difference highlights the impact of surgical techniques on the accuracy of tumor localization in colorectal surgeries. The recommendation for routine colonoscopic tattooing is driven by increased safety and reduced error rates, highlighting a clear preference for more reliable methods in surgical planning. In this study, although the errors in tumor localization during surgery or the effectiveness rates of the methods were not compared, the margin of error of colonoscopic surgery and what could be achieved were determined and are here stated. Nayor et al. performed tattooing on 20% of patients with colon cancer and stated that it increased colonoscopy accuracy rates during surgery [15]. El-Kefraoui et al. emphasized that erroneous bowel segments might be removed in patients without tattooing and may cause the prolongation of surgery time [29]. In our study, in two patients, because tumor localization could not be performed, colonoscopy was performed perioperatively, and the surgical strategy did not change compared with the preoperative period. Marking and tattooing in preoperative colonoscopy cannot solve the above-mentioned problems. The two patients in our study in whom tattooing was performed had early-stage cancer, and only laparoscopic cases where there was no palpation sensation were used. We do not think this procedure has an advantage other than facilitating the surgery by aiding in determining the patient’s tumor location. We believe that the aim of current research should be to plan the treatment of patients and increase their survival based on methods that allow for clear staging and tumor localization.

There are a few limitations to our study. First, it was retrospectively based. Second, we did not report on the pelvic floor types of our patients. Third, not all patients underwent control colonoscopy or rectosigmoidoscopy for tumor detection. We observed repeated endoscopies in patients in whom tumor localization errors were suspected. Another limitation of our study is that the pelvic types of male and female patients were not determined, and the comparative results were not given. Furthermore, given the importance of knowledge and awareness about the way in which the operation is performed and operational situations in the endoscopists and radiologists evaluating the tomography results, a limitation of our study is that, in exceptional cases, not all of the information obtained from the tomography and colonoscopy results checked immediately before the operation was recorded (for the study). The fact that our hospital was used as a pandemic hospital for approximately 1.5 years during the COVID-19 pandemic during the study period and that elective tumor cases were not admitted for approximately eight months after a major earthquake in our city is a final limitation of our study, as it limited the number of cases.

## 5. Conclusions

Studies are ongoing to increase the accuracy rates of sigmoid, rectosigmoid colon, and rectum tumor localization. To achieve high rates, all radiological, histopathological, and operational findings of prospective studies must be recorded, and new studies must be conducted. It is recommended that tomography evaluations involving this patient group be re-evaluated by the radiologist and the surgeon who is to operate. Due to the margin of error in rectosigmoid colon tumors, patients must be evaluated based on MRI, CT, and colonoscopy, together, as well as tumor localization performed by multidisciplinary units. 

## Figures and Tables

**Table 1:** Clinicopathological characteristics of patients.

	n (%)/Mean ± SD
Gender	
Female	17 (42.5)
Male	23 (57.5)
Ileus	4 (10.0)
Preoperative pathology	
Adenocarcinoma	39 (97.5)
High-grade dysplasia	1 (2.5)
Ileostomy during surgery	21 (52.5)
Receiving neoadjuvant chemotherapy	7 (17.5)
Pathological stage	
Rectum tm with complete response after neoadjuvant treatment	1 (2.5)
1	11 (27.5)
2A	18 (45.0)
3A	1 (2.5)
3B	6 (15.0)
3C	2 (5.0)
4C	1 (2.5)
Perioperative metastasis (peritoneal)	1 (2.5)
Type of surgery	
Anterior resection	7 (17.5)
Laparoscopic anterior resection	9 (22.5)
Laparoscopic low anterior resection	14 (35.0)
Low anterior resection	9 (22.5)
Total colectomy	1 (2.5)
Type of surgery technique	
Open	17 (42.5)
Laparoscopic	23 (57.5)
Largest tumor size (mm)	45.2 ± 20.8
Number of lymph nodes removed	22.3 ± 15.4
Lymphovascular invasion	16 (40.0)
Perineural invasion	8 (20.0)
Presence of tumor budding	6 (15.0)
Differentiation	
Unspecified	1 (2.6)
Poorly differentiated	1 (2.6)
Well differentiated	19 (48.7)
Moderately differentiated	18 (46.2)
Number of metastatic lymph nodes	1.4 ± 2.7
Distal surgical margin (mm)	45.1 ± 29.2
Previous surgery (related to existing tumor)	
Not	38 (95.0)
Loop ileostomy	1 (2.5)
Loop colostomy	1 (2.5)

SD: standard deviation.

**Table 2:** Tumor localization according to surgery, colonoscopy, preoperative CT, and retro CT.

	Surgery, n (%)	Preoperative Colonoscopy, n (%)	Preoperative CT, n (%)	Retro CT, n (%)
Sigmoid	16 (40.0)	18 (45.0)	14 (35.9)	19 (47.5)
Rectosigmoid	4 (10.0)	7 (17.5)	5 (12.8)	5 (12.5)
Rectum	20 (50.0)	15 (37.5)	12 (30.8)	16 (40.0)
No tumor seen or not reported	0 (0.0)	0 (0.0)	8 (20.5)	0 (0.0)

**Table 3:** Colonoscopy, preoperative CT, and retro CT localization accuracy rates according to surgery localization.

Surgery		Colonoscopy	Preoperative CT	Retro CT
Sigmoid				
	Sensitivity, %	93.8 (15/16)	75.0 (12/16)	100.0 (16/16)
	Specificity, %	87.5 (21/24)	87.5 (21/24)	87.5 (21/24)
Rectosigmoid				
	Sensitivity, %	50.0 (2/4)	50.0 (2/4)	75.0 (3/4)
	Specificity, %	86.1 (31/36)	91.7 (33/36)	94.4 (34/36)
Rectum				
	Sensitivity, %	75.0 (15/20)	60.0 (12/20)	80.0 (16/20)
	Specificity, %	100.0 (20/20)	100.0 (20/20)	100.0 (20/20)

**Table 4:** Comparison of accuracy rates of examination methods according to tumor region.

	Accuracy Rate, %			*p*
	Sigmoid	Rectosigmoid	Rectum	
Colonoscopy	93.8 (15/16)	50.0 (2/4)	75.0 (15/20)	0.084
Preoperative CT	75.0 (12/16)	50.0 (2/4)	60.0 (12/20)	0.554
Retro CT	100.0 (16/16)	75.0 (3/4)	80.0 (16/20)	0.140

**Table 5:** Relationships between individual and medical characteristics and surgical compliance.

		Accuracy Rate, %					
		Colonoscopy		Preoperative CT		Retro CT	
		n (%)	*p*	n (%)	*p*	n (%)	*p*
Gender	Female	15 (88.2)	0.428	13 (76.5)	0.191	16 (94.1)	0.373
	Male	17 (73.9)		13 (56.5)		19 (82.6)	
Ileus	No	28 (77.8)	0.566	23 (63.9)	0.999	31 (86.1)	0.999
	Yes	4 (100.0)		3 (75.0)		4 (100.0)	
Ileostomy during surgery	No	16 (84.2)	0.698	12 (63.2)	0.816	18 (94.7)	0.345
	Yes	16 (76.2)		14 (66.7)		17 (81.0)	
Open vs. laparoscopic	Open	14 (82.4)	0.999	12 (70.6)	0.524	16 (94.1)	0.373
	Laparoscopic	18 (78.3)		14 (60.9)		19 (82.6)	
Neoadjuvant CRT therapy	No	25 (75.8)	0.309	19 (57.6)	0.075	28 (84.8)	0.565
	Yes	7 (100.0)		7 (100.0)		7 (100.0)	
Lymphovascular invasion	No	20 (83.3)	0.690	16 (66.7)	0.999	22 (91.7)	0.373
	Yes	12 (75.0)		10 (62.5)		13 (81.3)	
Perineural invasion	No	26 (81.3)	0.650	21 (65.6)	0.999	28 (87.5)	0.999
	Yes	6 (75.0)		5 (62.5)		7 (87.5)	
Presence of tumor Budding	No	28 (82.4)	0.580	21 (61.8)	0.399	29 (85.3)	0.999
	Yes	4 (66.7)		5 (83.3)		6 (100.0)	

**Table 6:** Comparison of the accuracy rates of examination methods in all colon cancers and according to tumor location.

	Accuracy Rate, %			*p*
	Colonoscopy	Preoperative CT	Retro CT	
Sigmoid	93.8 (15/16)	75.0 (12/16)	100.0 (16/16)	0.110
Rectosigmoid	50.0 (2/4)	50.0 (2/4)	75.0 (3/4)	0.999
Rectum	75.0 (15/20)	60.0 (12/20)	80.0 (16/20)	0.450
All colon	80.0 (32/40)	65.0 (26/40)	87.5 (35/40)	0.049

Colonoscopy vs. preoperative CT, *p* = 0.133; colonoscopy vs. retro CT, *p* = 0.363; preoperative CT vs. retro CT, *p* = 0.018.

**Table 7:** Tumor findings of patients with tumor localization based on computed tomography and colonoscopy conflicting with the surgical findings.

Patients	Colonoscopy Result	Control Colonoscopy	Retro CT Result	Surgical Finding	Type of Surgery Performed
1	Rectosigmoid	Rectosigmoid	Sigmoid, 19–20 cm	Upper rectum	Lap. total mesorectal excision
2	Sigmoid	No	Sigmoid 21 cm	Rectosigmoid	Lap. low anterior resection involving upper rectum
3	Rectosigmoid	No	Middle rectum 8 cm	Middle rectum	Open total mesorectal excision
4	Sigmoid	No	Sigmoid 18 cm	Upper rectum	Open total mesorectal excision
5	Rectosigmoid	No	Sigmoid 19 cm	Sigmoid	Lap. anterior resection
6	Sigmoid	No	Rectosigmoid 18 cm	Rectosigmoid	Lap. low anterior resection involving upper rectum
7	Rectosigmoid	Rectosigmoid	Rectosigmoid 13 cm	Middle rectum	Lap. total mesorectal excision
8	Rectosigmoid	Rectosigmoid	Rectosigmoid 14 cm	Middle rectum	Lap. total mesorectal excision

## Data Availability

Data are unavailable due to privacy or ethical restrictions. However, we have data to be shared with the journal editor if the journal so requests.
